# Diagnosis of intracranial lesions using accelerated 3D T1 MPRAGE with wave-CAIPI technique: comparison with conventional 3D T1 MPRAGE

**DOI:** 10.1038/s41598-022-25725-x

**Published:** 2022-12-19

**Authors:** Eun Jung Lee, Min Gu Kim, Mi Sun Chung, Seon-Ok Kim, Jun Soo Byun, Younghee Yim

**Affiliations:** 1 Department of Radiology, Human Medical Imaging & Intervention Center, Seoul, Korea; 2 Department of Radiology, Chung-Ang University Hospital, Chung-Ang University College of Medicine, 102 Heukseok-Ro, Dongjak-Gu, Seoul, Republic of Korea; 3 Department of Clinical Epidemiology and Biostatistics, Asan Medical Center, University of Ulsan College of Medicine, 86 Asanbyeongwon-Gil, Songpa-Gu, Seoul, Republic of Korea

**Keywords:** Biological techniques, Biophysics, Neuroscience, Diseases

## Abstract

We aimed to evaluate the agreement in the diagnosis of intracranial lesions between conventional pre-contrast 3D T1 magnetization-prepared rapid gradient echo (MPRAGE) and wave-CAIPI (wave-controlled aliasing in parallel imaging) MPRAGE. Institutional review board approval was obtained and informed consent was waived for this retrospective study. We included 149 consecutive patients who had undergone brain MR with both conventional MPRAGE (scan time: 5 min 42 s) and wave-CAIPI MPRAGE (scan time: 2 min 44 s) from February to June 2018. All images were independently reviewed by two radiologists for the diagnosis of intracranial lesion and scored image quality using visual analysis. One technician measured signal-to-noise ratio. The agreement for diagnosis of intracranial lesion was calculated, and the intra- and interobserver agreements were analyzed by using kappa value. For the diagnosis of intracranial lesion, the conventional and wave-CAIPI MPRAGE demonstrated 99.7% of agreement (297 of 298) in the pooled analysis with very good agreement (k = 0.994). Intra- and inter-observer agreement showed very good (k > 0.9 in all) and good (k > 0.75) agreement, respectively. In the quantitative analysis, the signal-to-noise ratio had no difference (P > 0.05 for all). The overall image quality was poorer in images of wave-CAIPI MPRAGE (P < 0.001), but motion artifact had no difference between two sequences (P = 0.06). Compared to conventional MPRAGE, pre-contrast 3D T1 wave-CAIPI MPRAGE achieved higher agreement for the diagnosis of intracranial lesions and reduced the scan time by approximately 50%.

## Introduction

For the acquisition of 3D T1 weighted image (T1WI), magnetization-prepared rapid gradient echo (MPRAGE) technique has been widely used for high-resolution structural imaging due to clear contrast among gray matter, white matter and abnormal parenchymal lesions^1^. Moreover, 3D acquisition of MPRAGE permits tailored variable reconstruction plane for users in any directions, and resulted in relative reduction of overall scan time in patients who need multiple scan planes for proper diagnosis such as patients with neurodegenerative disease, congenital anomalies and epilepsy^1,2^. Other main applications of MPRAGE in neuroimaging are anatomical reference for other advanced MR imaging (MRI) data such as functional MRI and volumetric analysis^3^.

However, scan time for MPRAGE may be considerably long because generating T1 weighed contrast needs long inversion time before a gradient echo readout train and a recovery period^1^. Particular in cases of 1-mm isotropic resolution and whole brain coverage, total scan time might be over 10 min without parallel acquisition. Consequently, several problems related to long scan time could arise during the scanning of 3D T1 MRAGE, such as increased motion artifact, decreased patient’s compliance and more sedation in pediatric patients. Therefore, applying proper acceleration method is essential for the acquisition of 3D T1 MPRAGE to obtain images within practical scan time for clinical use.

Up to now, variable parallel imaging techniques such as sensitivity encoding (SENSE), generalized autocalibrating partially parallel acquisitions (GRAPPA), and controlled aliasing in parallel imaging results in higher acceleration (CAIPIRINHA) have been routinely used to reduce scan time in MPRAGE by reducing the number of phase-encoding steps using coil sensitivity encoding from multichannel receiver arrays^4–6^. However, known parallel acquisition techniques are limited in their ability to increase acceleration factors in daily practice because as the parallel factor increases, the image reconstruction quality becomes degraded by the noise amplification induced by the increased geometric (g)-factor and the acquisition of fewer data points^7^. To overcome these problems, the wave-CAIPI (wave-controlled aliasing in parallel imaging) technique was recently suggested^8^. This technique simultaneously generates the sinusoidal shape of Gy and Gz gradients with a π/2 phase shift between two waveforms during the frequency-encoding step and consequently creates a staggered corkscrew trajectory for k-space sampling. This k-space filling strategy paved the way for fast 3D acquisition with low artifacts and negligible g-factor penalties by spreading the aliasing evenly in all spatial directions^8^. Wave-CAIPI acceleration technique had been applied on SWI, quantitative susceptibility mapping, rapid acquisition with refocusing echoes, and MPRAGE sequences^8–13^. Recent study revealed that volume measurement using wave-CAIPI MPRAGE clinically comparable to that of MPRAGE GRAPPA^14^. Also, recent studies applying wave-CAIPI MPRAGE to pediatric subjects showed substantial reduction of scan time and reliable diagnostic performance^15,16^. However, despite significant benefit on making the scan time shorter, the diagnostic performance of intracranial lesions using pre-contrast wave-CAIPI MPRAGE has not been evaluated based on large number of clinical data and comparisons of diagnostic performance between pre-contrast conventional MPRAGE and wave-CAIPI MPRAGE have not been conducted.

We therefore aimed to investigate the agreements for the diagnosis of intracranial lesions between conventional pre-contrast 3 D T1WI MPRAGE without wave-CAIPI acceleration and wave-CAIPI MPRAGE. We also compared values of quantitative parameters image quality in both sequences.

## Materials and methods

### Patients

**Figure 1:**
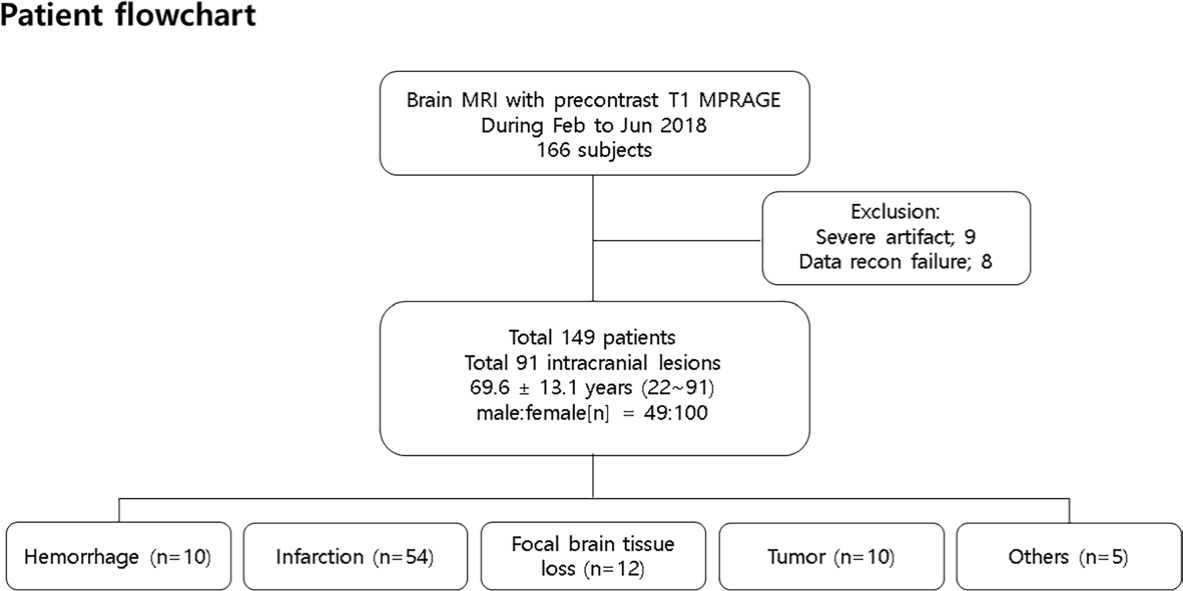
Patient flow chart.

This retrospective study included 149 consecutive patients who had undergone brain MRI with pre-contrast 3D T1 MPRAGE from February to June 2018 in a single referral center (Fig. 1). The inclusion criteria for this study were as follows: (1) patients who underwent MR for the evaluation of intracranial lesions, (2) patients who scanned both sequences as pre-contrast 3D T1 MPRAGE without wave-CAIPI acceleration (conventional MPRAGE) and with wave-CAIPI acceleration (wave-CAIPI MPRAGE), and (3) patient age > 20 years. Exclusion criteria were as follows: (1) MR images with severe motion or metal artifacts and (2) data reconstruction failure. We retrospectively collected demographic and clinical data, including age, sex, and past medical history of all the subjects by reviewing their electronic medical records.

All procedures performed in studies involving human participants were in accordance with the ethical standards of the institutional and/or national research committee and with the 1964 Helsinki declaration and its later amendments or comparable ethical standards. Institutional review board approval was obtained and informed consent was waived for this retrospective study (IRB No.: Chung-Ang University Hospital, 1821-012-362). We have reported the methods and results in accordance with the STROBE (Strengthening the reporting of observational studies in epidemiology) guidelines^17^.

### Image acquisition

All the MRI examinations were performed by using two 3.0 T MRIs (Magnetom Skyra, SIEMENS, Erlangen, Germany), with a dedicated 64 channel head & neck coil under the IDEA environment. The MR scan parameters of conventional and wave-CAIPI MPRAGE are shown in detail in Table 1. Scan parameters of conventional 3D T1 MPRAGE was based on the commercially released and clinically used parameters while parameters of wave-CAIPI MPRAGE were modulated and optimized based on previously published formula^8,11^. The total acquisition times were 5 min 42 s for conventional MPRAGE and 2 min 44 s for wave-CAIPI MPRAGE.

**Table 1:** Image parameters.

	Conventional MPRAGE	Wave-CAIPI MPRAGE
Field of view (phase × frequency, mm)	256 × 256	256 × 256
Voxel size (phase × frequency × slice, mm)	1 × 1 × 1	1 × 1 × 1
TR (ms)	2500	2500
TE (ms)	2.98	3.06
TI (ms)	1100	1100
Number of slices	176	192
Parallel imaging method	GRAPPA	CAIPIRINHA
Acceleration factor PE (phase encoding direction)	2	2
Acceleration factor 3D (slice encoding direction)	–	2
Scan time	5 min 42 s	2 min 44 s

*CAIPIRINHA* controlled aliasing in parallel imaging results in higher acceleration, *GRAPPA* generalized autocalibrating partially parallel acquisitions, *MPRAGE* magnetization-prepared rapid gradient echo, *TE* echo time, *TI* inversion time, *TR* repetition time, *wave-CAIPI* wave-controlled aliasing in parallel imaging.

### Image analysis

All MRIs were independently reviewed by two neuroradiologists (8 years and 7 years of experience, respectively). All cases were randomized and anonymized in each review session and raters were blinded for which sequence was being reviewed in each session. Each observer independently performed the diagnosis with a 2-week interval to prevent recall bias; both observers were blinded to the results of the other observer and other sequences (conventional MPRAGE and wave-CAIPI MPRAGE). Diagnoses of intracranial lesions were divided into six categories: (1) no lesion, (2) hemorrhage (all stages), (3) infarction (acute and old territorial and old lacunar infarctions), (4) focal brain tissue loss with uncertain cause (except previous hemorrhage or infarction), (5) tumor, and (6) other diseases. In patients with multiple pathologies, up to three different categories were diagnosed in a single patient.

Following the diagnosis of intracranial lesions, the quantitative image quality (signal-to-noise ratio [SNR] and SNR_GM-WM_) and subjective image quality were evaluated. SNR and SNR_GM-WM_ were measured by one author using a dedicated workstation (TeraRecon, ver. 4.4.12, Foster City, USA). The signal intensity and standard deviation (SD) of the brain parenchyma at three levels of brain parenchyma as follows: (a) the white and gray matter of the centrum semiovale (ROI size, about 10 mm^2^); (b) the center of the pons (ROI size > 40 mm^2^); and (c) the white matter of the cerebellar hemisphere (ROI size > 40 mm^2^). The SNR was defined as 0.695 × (signal intensity)/(noise), with noise measured as SD of the parenchyma at the level of centrum semiovale (ROI area > 200 mm^2^)^18,19^. We did not directly measure the noise in the surrounding air because of the inhomogeneous noise distribution induced by parallel acquisition^20^. Therefore, we measured the SD of the brain parenchyma. The SNR_GM-WM_ at the level of centrum semiovale was calculated using the following formula: SNR_GM-WM_ = SNR_GM_ − SNR_WM_.

The image qualities (overall image quality and motion artifact) of both conventional and wave-CAIPI MPRAGE were graded by two neuroradiologists using a five-level scale based on visual analysis. The points on the scale were defined as follows: 1 = non-diagnostic image quality due to strong artifacts; 2 = severe blurring that resulted in significant limitation in evaluation; 3 = moderate blurring that slightly compromised assessment; 4 = slight blurring that did not compromise image assessment; and 5 = excellent image quality without artifacts.

### Statistical analysis

The summary statistics are presented as numbers and percentages for categorical variables and means with standard deviations for continuous variables. The agreement for the diagnosis of intracranial lesions between conventional and wave-CAIPI MPRAGE was calculated using kappa values in the pooled analysis for observer 1 and 2, and its two-sided 95% confidence interval (CI) was estimated. The intra- and inter-observer agreement for the diagnosis and presence of intracranial lesions was analyzed using kappa values. The kappa results were interpreted as being in poor (0–0.20), fair (0.21–0.40), moderate (0.41–0.60), good (0.61–0.80), or very good (0.81–1) agreement^21^. The SNR, SNR _GM-WM_, and image quality were compared using the paired T-test. Wilcoxon signed rank test were used for the comparison of image qualities. All statistical analyses were performed using MedCalc, version 15.0 (MedCalc Software, Ostend, Belgium) or SPSS software (version 20.0; SPSS, Chicago, IL), with one-sided *P* values < 0.05 considered to be statistically significant.

## Results

The mean age of the 149 patients was 69.6 ± 13.1 years (range 22–91 years and male:female [n] = 49:100).

**Figure 2:**
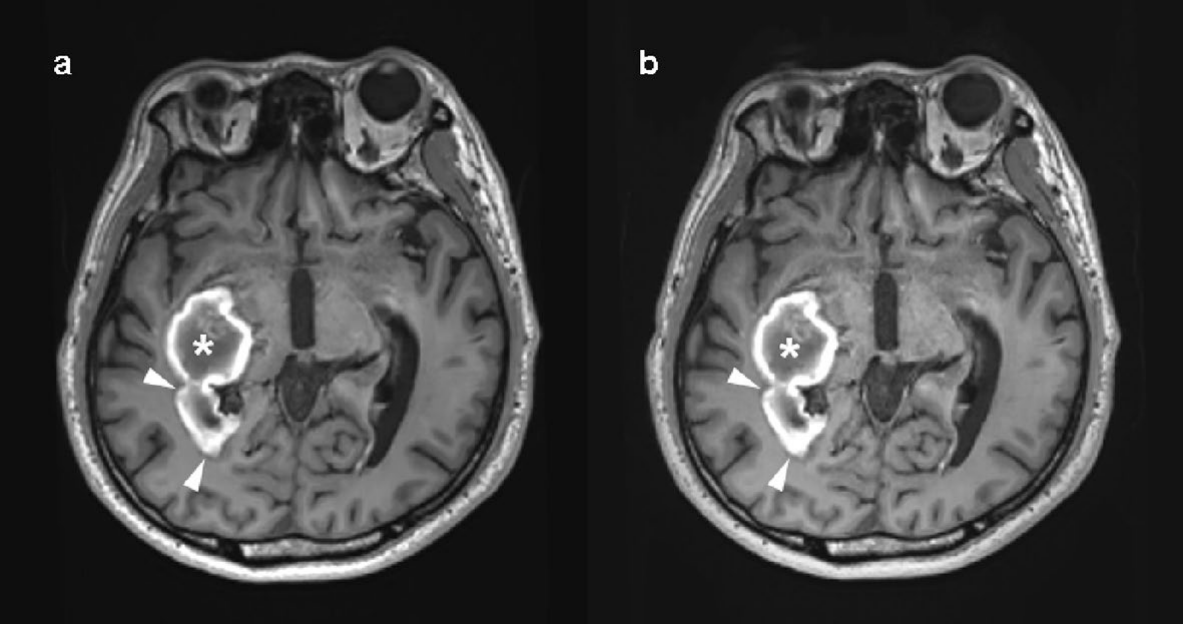
Diagnosis of hemorrhage using conventional and wave-CAIPI MPRAGE. Both conventional pre-contrast 3D T1 MPRAGE (**a**) and wave-CAIPI MPRAGE (**b**) clearly showed subacute hemorrhage in the right basal ganglia (asterisk) extending into the right lateral ventricle, forming intraventricular hemorrhage (arrowhead). *MPRAGE* magnetization-prepared rapid gradient echo, *wave-CAIPI* wave-controlled aliasing in parallel imaging.

**Figure 3:**
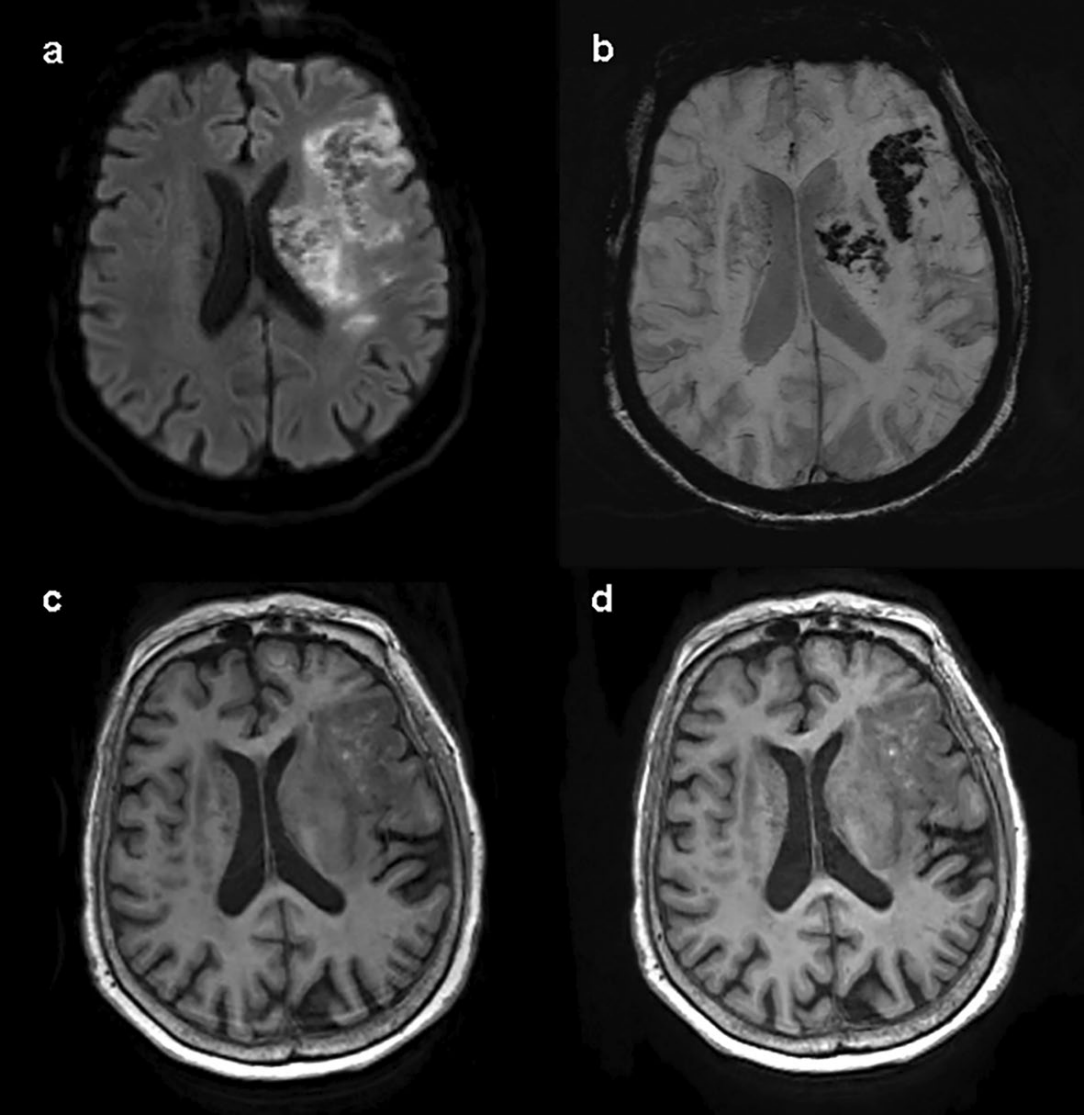
Subacute infarction on conventional and wave-CAIPI MPRAGE. An 87-year-old woman with right-sided weakness 7 days ago. Subacute stage infarction with hemorrhagic transformation in the left middle cerebral artery territory diagnosed on diffusion-weighted image (**a**) and susceptibility-weighted image (**b**). Both conventional pre-contrast 3D T1 MPRAGE (**c**) and wave-CAIPI MPRAGE (**d**) show parenchymal swelling with several tiny hyperintense dots suggesting hemorrhagic transformation foci in the left middle cerebral artery territory. *MPRAGE* magnetization-prepared rapid gradient echo, *wave-CAIPI* wave-controlled aliasing in parallel imaging.

**Figure 4:**
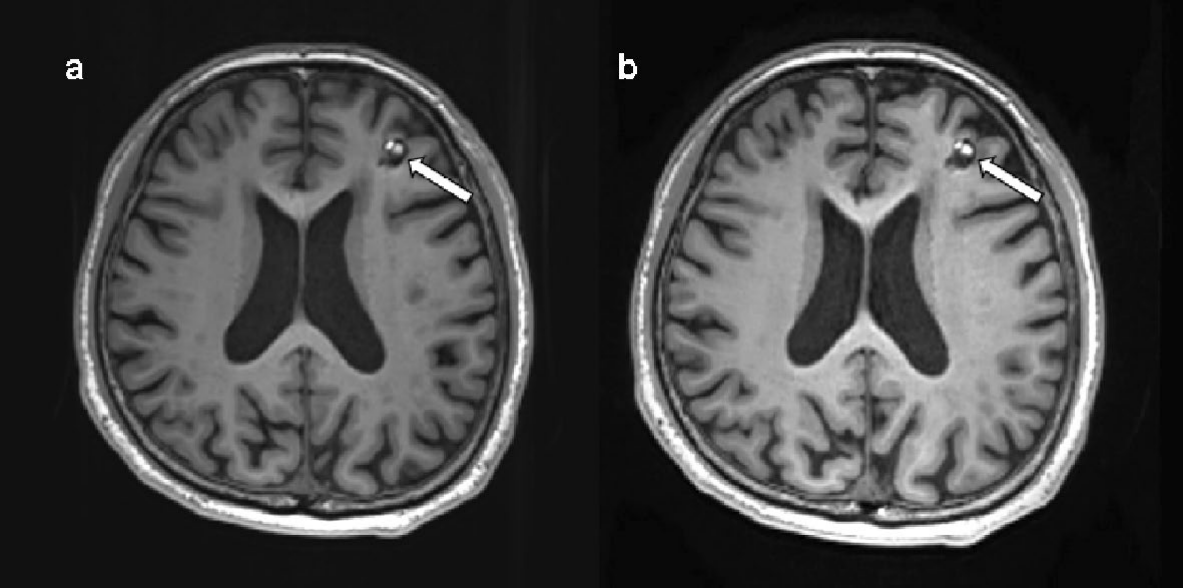
Cavernous malformation on conventional and wave-CAIPI MPRAGE. A 64-year-old man who had an approximately 1-cm characteristic popcorn-shaped mass with T1 high signal intensity and hemosiderin rim in the left frontal lobe, suggestive of cavernous malformation. It is well demarcated on both conventional pre-contrast 3D T1 MPRAGE (**a**) and wave-CAIPI MPRAGE (**b**). *MPRAGE* magnetization-prepared rapid gradient echo, *wave-CAIPI* wave-controlled aliasing in parallel imaging.

For the diagnosis of intracranial lesions, conventional and wave-CAIPI MPRAGE demonstrated 99.7% agreement (297 of 298) in the pooled analysis with very good agreement (k = 0.994, Table 2). In terms of the presence or absence of intracranial lesions, both sequences showed 100% agreement. In total, 91 lesions from 65 patients were diagnosed using both conventional and wave-CAIPI MPRAGE. The diagnoses included 10 hemorrhages (two subarachnoid hemorrhages, two subdural hemorrhages, two intracranial hemorrhages, one intraventricular hemorrhage, and three old hemorrhage sequelae, Fig. 2), 54 infarctions (46 lacunar infarctions, seven old infarctions, and one subacute infarction, Fig. 3), 12 focal brain tissue loss, 10 tumors (six intra-axial masses and four extra-axial masses, Fig. 4), and five other diseases (three benign cystic lesions, such as arachnoid cysts and hippocampal cysts, one corpus callosum agenesis, and one multiple system atrophy). Intra-observer and inter-observer agreements in conventional and wave-CAIPI MPRAGE similarly showed very good (k > 0.9 in all) and good (k > 0.75) agreement (Table 3). In quantitative analysis, SNR at all levels (centrum semiovale, pons, and cerebellum) and SNR_GM-WM_ of conventional and wave-CAIPI MPRAGE did not show any difference (P > 0.05 for all, Table 4). The overall image qualities of wave-CAIPI MPRAGE and of conventional MPRAGE were statistically different (median [interquartile range; range], 5 [5–5; 3–5] for conventional MPRAGE and 5 [5–5; 2–5] for wave-CAIPI MPRAGE, P < 0.001). However, the motion artifact of wave-CAIPI MPRAGE and of conventional MPRAGE had no difference (median [interquartile range; range], 5 [5–5; 4–5] for conventional MPRAGE and 5 [5–5; 3–5] for wave-CAIPI MPRAGE, P = 0.06).

**Table 2:** Agreement for detecting intracranial lesion between conventional and wave-CAIPI MPRAGE.

	Percent agreement (%, number of agreement/total)	Kappa value (95% CI)
Overall	99.7 (297/298)	0.994 (0.984–1.000)
Observer 1	99.3 (148/149)	0.988 (0.967–1.000)
Observer 2	100.0 (149/149)	1.000 (1.000–1.000)

*CI* confidence interval, *MPRAGE* magnetization-prepared rapid gradient echo, *wave-CAIPI* wave-controlled aliasing in parallel imaging.

**Table 3:** Intraobserver and interobserver agreement between conventional and wave-CAIPI MPRAGE.

	Imaging methods	Kappa value (95% CI)
**Intraobserver agreement**		
Presence of intracranial lesion	Conventional	0.945 (0.891–1.000)
	Wave-CAIPI	0.959 (0.912–1.000)
Diagnosis of target lesion	Conventional	0.911 (0.852–0.970)
	Wave-CAIPI	0.923 (0.867–0.978)
**Interobserver agreement**		
Presence of intracranial lesion	Conventional	0.762 (0.657–0.868)
	Wave-CAIPI	0.762 (0.657–0.868)
Diagnosis of target lesion	Conventional	0.762 (0.669–0.855)
	Wave-CAIPI	0.762 (0.669–0.855)

*CI* confidence interval, *MPRAGE* magnetization-prepared rapid gradient echo, *wave-CAIPI* wave-controlled aliasing in parallel imaging.

**Table 4:** Comparison of signal to noise ratio between conventional and wave-CAIPI MPRAGE.

	Level	Conventional MPRAGE (range)	Wave-CAIPI MPRAGE (range)	P-value
SNR	Centrum semiovale	21.1 ± 8.6 (6.3–44.3)	20.6 ± 4.2 (11.6–33.1)	0.57
	Pons	12.9 ± 4.0 (6.9–21.3)	12.9 ± 2.5 (8.8–22.0)	0.88
	Cerebellum	15.3 ± 5.2 (8.6–29.4)	15.4 ± 3.9 (7.8–26.8)	0.87
SNR_GM-WM_	Centrum semiovale	6.8 ± 3.3 (1.9–17.8)	6.2 ± 2.4 (2.2–12.4)	0.06

*MPRAGE* magnetization-prepared rapid gradient echo, *GM* grey matter, *WM* white matter, *SNR* signal to noise ratio, *wave-CAIPI* wave-controlled aliasing in parallel imaging.

## Discussion

Compared with conventional MPRAGE, wave-CAIPI MPRAGE achieved excellent agreement for the diagnosis of various intracranial lesions and reduced the scan time by approximately 50% in this study. In addition, the SNR and SNR_GM-WM_ of wave-CAIPI MPRAGE did not differ significantly from those of conventional MPRAGE. Subjective image quality of wave-CAIPI MPRAGE was poorer, however, motion artifact is comparable between two sequences. Thus, we suggest that pre-contrast 3D T1 wave-CAIPI MPRAGE is a reliable fast scan method that could be an alternative to conventional 3DT1 MPRAGE in our routine practice.

Acquiring appropriate MR images with a high acceleration factors is often challenging, because augmented g-factor from the summation of aliased voxels resulted in noise amplification^22^. Therefore, aliasing control is essential for fast MR imaging. The corkscrew k-space trajectory of wave-CAIPI technique achieves both a highly efficient k-space sampling and maximization of the distance between aliased voxels in all spatial dimensions (x, y, and z)^8,9^. In addition, for the MPRAGE acquisition, wave-CAIPI MPRAGE utilized a new reordering scheme for further speed up^11^. 3D MPRAGE sequences needs two steps for the acquisition of full k-space; (1) the inner loop consists of magnetization preparation, rapid gradient echo acquisition of a kx–kz plane, and subsequent recovery period, and (2) the outer loop repeats the inner loops along whole ky plane to generate 3 dimensions^1^. Proposed reordering scheme in wave-CAIPI MPRAGE took advantage of both inner loop acceleration (interleaved scan) and outer loop acceleration (decreased number of inner loop cycles) for the efficient shortening of the scan time^11^. Here, we demonstrated wave-CAIPI MPRAGE achieves proper diagnostic performance for intracranial lesions within a half of scan time to obtain enough clinical information and make decisions even though subjective image quality is relatively poorer than conventional MPRAGE.

On the other hands, wave-CAIPI MPRAGE could have several drawbacks in clinical applications. First, previous technical report mentioned that the motion artifacts could be worsen in images using the wave-CAIPI technique^8^. However, motion artifacts between two sequences were comparable in this study. We presumed that shorter scan time of wave-CAIPI MPRAGE reduced chance to meet patient motions during MRI scanning. The actual clinical implication of wave-CAIPI based sequences in patients with higher probability of motion artifacts should need further clinical study. Second, the overall image qualities of wave-CAIPI MPRAGE were lower compared with conventional MPRAGE, even though SNR were comparable in both sequences. Twice higher acceleration factor of wave-CAIPI could affect noise amplification and less overall image quality compared to those of conventional MPRAGE. However, further development of reconstruction algorithms of wave-CAIPI MPRAGE such as iterative reconstruction, reconstruction filters and surface coil combination could be helpful for the improvement of overall image quality^23^. Third, wave-CAIPI MPRAGE needs more computational power for the reconstruction of images from fewer data points. Furthermore, applying well-designed multi-channel coil system could take a full advantage of the higher acceleration of wave-CAIPI sequences.

Our study had several limitations to note. First, selection bias could have existed because this study underwent at a single referral center with a relatively small number of patients. Second, even though we blinded related clinical or sequence information and interpreted the image independently in random manner, there might exist inevitable recall bias or memory effect that we could not control. This might affect not only the result of agreement in diagnostic performance between two different sequences, but also intra-observer agreement. The inter-rater agreement was relatively low between two readers, and the different imaging interpretation experience might have affected the result. We expect that taking longer interval in image interpretation or more systematic pre-interpretation session could be helpful to prevent possible bias in agreement. Third, we focused on the diagnostic performance of intracranial lesions in this study. Therefore, other clinical applications of wave-CAIPI MPRAGE, such as volumetric analysis and post-contrast 3D T1 MPRAGE, were not fully evaluated. For the validation of new sequences, accessing diagnostic performances of intracranial lesions of new sequences should be an essential and basic step before the further complex analysis such as volumetric analysis. Based on our results, attempts for further studies of variable clinical applications and the optimization of scan parameters in each sequence of wave-CAIPI MPRAGE could be supported. Fourth, we didn’t compare the sequences with perfectly same acceleration factors or slice numbers. We had to adjust these because we experienced inevitable technical errors when adapting high acceleration numbers to conventional 3D T1 MPRAGE and when adapting same slice numbers to wave-CAIPI MPRAGE. Since our main purpose is to find out the best way to save the scan time as much as maintaining the original quality of the image, we adopted numbers that best visualize the image. Fifth, even though pre-contrast MRI has several advantages compared to using contrast media such as reducing risk of adverse events or reducing cost or preparation time for scanning by not securing the intravenous line, checking kidney function or preparing contrast media, post-contrast study is sometimes inevitable in certain clinical circumstances such as diagnosing intracranial tumors or inflammation^24^. In this perspective, although our study resulted good performance in diagnosing various intracranial lesions with pre-contrast image, further evaluation and validation should be followed to apply wave-CAIPI MPRAGE to variety of clinical use. Sixth, this study was based on probable diagnoses considering image findings, and conclusive diagnoses with pathologic confirmation were not acquired. Because of the lack of pathologic specimens, we performed an agreement study with conventional MPRAGE and wave-CAIPI MPRAGE rather than a diagnostic accuracy study.

## Conclusion

In conclusion, 3D pre-contrast wave-CAIPI MPRAGE is a reliable method for the diagnosis of intracranial lesions within half the scan time of conventional MPRAGE without the wave-CAIPI acceleration in case of post-contrast study is not required for diagnosis. Considering the reduced scan time and preservation of diagnostic performance, 3D pre-contrast wave-CAIPI MPRAGE could be a good option to substitute conventional MPRAGE in daily practice.

## Data Availability

The datasets used and/or analysed during the current study available from the corresponding author on reasonable request, but there could be certain process to release the data due to the local policy.

## Abbreviations

MPRAGE: Magnetization-prepared rapid gradient echo
wave-CAIPI: Wave-controlled aliasing in parallel imaging
